# An engineered multicomponent bone marrow niche for the recapitulation of hematopoiesis at ectopic transplantation sites

**DOI:** 10.1186/s13045-016-0234-9

**Published:** 2016-01-25

**Authors:** Mónica S. Ventura Ferreira, Christian Bergmann, Isabelle Bodensiek, Kristina Peukert, Jessica Abert, Rafael Kramann, Paul Kachel, Björn Rath, Stephan Rütten, Ruth Knuchel, Benjamin L. Ebert, Horst Fischer, Tim H. Brümmendorf, Rebekka K. Schneider

**Affiliations:** Department of Hematology, Oncology, Hemostaseology and Stem Cell Transplantation, University Hospital Aachen, RWTH Aachen University, Aachen, Germany; Institute of Pathology, University Hospital Aachen, RWTH Aachen University, Aachen, Germany; Department of Dental Materials and Biomaterials Research, University Hospital Aachen, RWTH Aachen University, Aachen, Germany; Department of Clinical Immunology and Nephrology, University Hospital Aachen, RWTH Aachen University, Aachen, Germany; Renal Division, Department of Medicine, Brigham and Women’s Hospital, Harvard Medical School, Boston, MA USA; Department of Orthopaedic Surgery, University Hospital Aachen, RWTH Aachen University, Aachen, Germany; Electron Microscopy Facility, University Hospital Aachen, RWTH Aachen University, Aachen, Germany; Division of Hematology, Department of Medicine, Brigham and Women’s Hospital, Harvard Medical School, Boston, MA USA; Broad Institute of Harvard University and Massachusetts Institute of Technology, Cambridge, MA USA

## Abstract

**Background:**

Bone marrow (BM) niches are often inaccessible for controlled experimentation due to their difficult accessibility, biological complexity, and three-dimensional (3D) geometry.

**Methods:**

Here, we report the development and characterization of a BM model comprising of cellular and structural components with increased potential for hematopoietic recapitulation at ectopic transplantation sites. Cellular components included mesenchymal stromal cells (MSCs) and hematopoietic stem and progenitor cells (HSPCs). Structural components included 3D β-tricalcium phosphate (β-TCP) scaffolds complemented with Matrigel or collagen I/III gels for the recreation of the osteogenic/extracellular character of native BM.

**Results:**

In vitro, β-TCP/Matrigel combinations robustly maintained proliferation, osteogenic differentiation, and matrix remodeling capacities of MSCs and maintenance of HSPCs function over time. In vivo, scaffolds promoted strong and robust recruitment of hematopoietic cells to sites of ectopic transplantation, vascularization, and soft tissue formation.

**Conclusions:**

Our tissue-engineered BM system is a powerful tool to explore the regulatory mechanisms of hematopoietic stem and progenitor cells for a better understanding of hematopoiesis in health and disease.

**Electronic supplementary material:**

The online version of this article (doi:10.1186/s13045-016-0234-9) contains supplementary material, which is available to authorized users.

## Background

The different components of the bone marrow (BM) microenvironment—consisting of (a) hematopoietic cells, (b) stromal cells and vasculature, (c) extracellular matrix, and (d) bone—are critical to explore for a better understanding of hematopoiesis during health and disease. These components are often inaccessible for controlled and rapid experimentation, thus limiting studies to the evaluation of conventional cell culture and transgenic animal models. The rationale to develop ectopic transplantable BM niches arises from the need to dissect regulatory mechanisms in the BM and the hematopoietic-stroma interaction. So far, no gold standard exists to specifically analyze the role of the BM stroma in vivo or to genetically modify stroma in its natural environment as stroma is not sufficiently transplantable in contrast to hematopoietic cells [1, 2].

Few approaches including in vivo imaging [3, 4], the design of three-dimensional (3D) environments using biomaterials [5–10], and BM-on-a-chip [11] for the study of hematopoiesis have been introduced to date, but these system lack full BM recreation, as hematopoietic stem and progenitor cell (HSPC) interaction with the endosteal niche or with the supporting stroma is compromised or simply the geometry beneficial for a controlled manipulation is still missing.

Bioceramics such as β-tricalcium phosphate (β-TCP) are particularly interesting for bone tissue engineering as they provide characteristics for cellular interactions while ensuring superior biomechanical properties [12]. Matrigel is a basement membrane protein mixture typically used in vivo to stimulate tissue formation. [8]. Here, we combined 3D β-TCP scaffolds with defined and controlled geometry (bone component) with an extracellular matrix component composed of either collagen I/III or Matrigel (matrix component) to establish co-cultures of HSPCs and mesenchymal stromal cells (MSCs) (cellular component). The ultimate goal of the current study is to create artificial, transplantable BM niches that support hematopoiesis while allowing for the genetic modification of both hematopoietic and mesenchymal cells as to dissect their interaction.

## Methods

### β-TCP scaffolds

β-TCP scaffolds were fabricated using slip casting into 3D-printed wax molds. First, two virtual models were constructed using computer-aided design (3-matic, Materialise, Leuven, Belgium). The models had a cylindrical shape with an inner diameter of 9.6 mm and a height of 4.9 mm. A rectangular lattice with 500-μm struts was incorporated into one of the models. The struts had a spacing of 2 mm and were connected to the cylinder. Into the second virtual model, a lattice with 800-μm struts (spacing 2.5 mm) was incorporated in the same way. Finally, a sprue with a diameter of 9.6 mm and a height of 2.1 mm was added on one side of the cylinders. Both models were printed using a 3D wax printer (T76®PLUS, Solidscape, Idar-Oberstein, Germany) to generate the wax molds for the slip casting process. A suspension consisting of 68.7 wt% β-TCP, 29.3 wt% distilled water, and 2 wt% organic additives (0.2 wt% Contraspun, 1.4 wt% Optapix, 0.4 wt% Dolapix, Zschimmer und Schwarz, Lahnstein, Germany) was synthesized. The suspension was homogenized for 30 s using a SpeedMixer^TM^, (DAC 150.1 FVZ, Hauschild, Hamm, Germany) at a mixing rate of 3000 rpm. Afterwards, the suspension was filled with a pipette into the wax molds. The filled molds were devolatilized in a desiccator, and the suspension within was dried for 24 h at room temperature. The sprue was cut off with a scalpel until the ends of the vertical wax struts were exposed. The samples were heat treated for 30 min at 105 °C to melt out the wax (heating rate 2.5 K/min) and subsequently sintered for 3 h at 1200 °C (heating rate 3 K/min). The generated β-TCP scaffolds were cleaned in an ultrasound bath to remove particulate matter and dried at 80 °C for 24 h. Finally, the scaffolds were sterilized by autoclaving and dried at 80 °C for 24 h before they were used for cell culture.

### Collagen I/III gels and Matrigel®

Collagen I/III were produced as previously described [13–16], and human mesenchymal stromal cells (hMSCs) or murine BM-derived mesenchymal stem cells (mBMSCs) were seeded at a density of 1 × 10^6^ cells/mL. Matrigel® basement membrane matrix complex (BD Biosciences, 354234) was handled according to the manufacturer’s instructions, and mBMSCs were seeded at a density of 1 × 10^6^ cells/mL. Two hundred microliters of either collagen I/III gel or Matrigel® was combined with each one β-TCP scaffold. Gel polymerization was achieved by 1-h incubation at 37 °C in a 20 %-O_2_/5 %-CO_2_-humidified atmosphere.

### Isolation and culture of hMSCs

MSCs from human femoral head spongiosa were collected after hip replacement surgeries and followed approved guidelines of the Ethics Committee of RWTH Aachen University. Cell isolation was performed according to previously described protocols [13–16]. For all experiments, hMSCs were passaged between 2–4 times before use. hMSCs were seeded on the β-TCP scaffolds at a density of ca. 1.5 × 10^5^ cells/scaffold. Osteogenic differentiation was performed according to published protocols [8, 14].

### Isolation and culture of human CD34^+^ progenitors

CD34^+^ progenitors were obtained by immunomagnetic bead selection (Miltenyi Biotec, Bergisch-Gladbach, Germany) from human umbilical cord blood units collected according to the guidelines of the Ethics Committee of RWTH Aachen University (EK187/08). CD34^+^ progenitors were cultured in StemSpan serum-free medium in the presence of SCF (50 ng), TPO (20 ng), Flt3-L (50 ng), and IL-6 (10 ng, all Peprotech, London, UK) as before [7, 8].

### Isolation and culture of mBMSCs

mBMSCs were isolated from BM aspirates of 6–8-week-old C57BL/6 mice by mechanical crushing and collagenase treatment to liberate the stromal cells from the endosteal bone as described before [17]. Cells from six pooled mice were used for each experiment. mBMSCs were expanded in DMEM/F-12 with Glutamax™ (Life Technologies) supplemented with 1 nM dexamethasone (Sigma, Steinheim, Germany), 1 ng/mL fibroblast growth factor-2 (Peprotech, London, UK), 5 ng/mL epidermal growth factor (Peprotech), 2 % fetal calf serum (PAN Biotech), and 2 % penicillin/streptomycin (Life Technologies). mBMSCs were used between passages 2–4 for seeding.

### Isolation and culture of murine c-kit^+^ cells

The hematopoietic cell fraction was collected by mechanical crushing/flushing of long bones and the pelvis, and c-kit^+^ cells were isolated by immunomagnetic bead selection using mouse CD117 (c-kit) microbeads (Miltenyi Biotec, Bergisch-Gladbach, Germany). Purity was confirmed by flow cytometry. Freshly isolated c-kit^+^ cells were used for co-cultures at ca. 1.5 × 10^5^ cells/well and maintained in StemSpan serum-free expansion medium (SFEM) (Stem Cell Technologies Inc., Vancouver, Canada) containing 50 ng/mL stem cell factor (Peprotech), 50 ng/mL thrombopoietin (Peprotech), and 1 % penicillin/streptomycin (Life Technologies).

### Culture conditions in β-TCP scaffolds

β-TCP scaffolds were transferred to 48-well tissue culture plates (Corning, Wiesbaden, Germany), and hMSCs embedded in the collagen I/III gels or directly seeded on the β-TCP scaffolds were kept in culture for 8 weeks. mBMSCs embedded in gels were pre-expanded for 1 week, and c-kit^+^ cells were seeded and co-cultures kept at 37 °C in a 20 %-O_2_/5 %-CO_2_-humidified atmosphere. Cells were recovered by vigorous pipetting and subsequent gel digestion using collagenase II (Life Technologies) for collagen I/III gels and dispase II (Life Technologies) for Matrigel® gels.

### Flow cytometry

For analysis of maintenance of primitive phenotype in the human setting, 5-day cultured CD34^+^-isolated cells co-cultured with hMSCs for 5 days in β-TCP scaffolds with or without matrix component were assessed for the combined expression of CD34-FITC and CD38-PE (Miltenyi Biotec). For analysis of primitive phenotype and differentiation ability in the murine setting, c-kit^+^ cells from in vitro co-cultures were recovered at days 4, 7, 10, 14, and 18. In the in vivo setup, cells were recovered from explanted scaffolds 4 and 8 weeks after transplantation. Antibodies CD117-allophycocyanin, Sca1-phycoerythrin, lineages (CD3, CD5, B220, G1, CD11b, Ter119)-eFluor®450, Gr-1-allophycocyanin-cyanine7, CD11b-allophycocyanin, CD19-phycoerythrin-cyanine7, CD3-phycoerythrin, and Ter119-peridinin chlorophyll-cyanine5.5 (all eBiosciences, Frankfurt, Germany) were used at 1:100, except for lineage antibodies used at 1:200 as recommended, in PBS/1 %-FCS for 30 min at 4 °C. A minimum of 100,000 events were acquired on a FACSCantoII flow cytometer (Becton Dickinson). Data were further analyzed using FlowJo (Tree Star Inc., Ashland, USA).

### Methylcellulose colony assays

C-kit^+^ cells derived from 4-day or 14-day cultures were plated at 1 × 10^5^ cells per 35-mm petri dish in duplicates of serum-free methylcellulose medium (MethoCult GF M3434, Stem Cell Technologies Inc., Vancouver, Canada). After 12 days of incubation at 37 °C and 5 % CO_2_ in a humidified atmosphere, colonies were scored using an inverted light microscope (Leica, Wetzlar, Germany). Colony-forming unit granulocyte-macrophages (CFU-GM) and colony-forming unit granulocyte-erythrocyte-macrophage-megakaryocyte (CFU-GEMM) were assayed according to morphological criteria.

### SEM and FESEM in cryo-mode

β-TCP scaffolds from in vitro cultures and in vivo explantations were prepared for scanning electron microscopy (SEM) by fixation in 3 % glutaraldehyde for at least 24 h at 4 °C. A graded ethanol series of 30, 50, 70, 90, and 100 % followed for sample dehydration. Sample preservation was achieved by hexamethyldisilizane (HMDS) drying. Before gold sputtering and fixation on SEM stubs, scaffolds were sectioned one time longitudinal and orientated in the cross-sectional area for visualization. A field emission SEM microscope (ESEM XL 30 FEG, FEI, Philips, Eindhoven, The Netherlands) with a high-vacuum environment was used.

For a more detailed analysis of gel structure, field emission scanning electron microscopy (FESEM) in cryo-mode was performed for collagen I/III and Matrigel®. Gels were rapidly frozen in liquid nitrogen and transferred to the high-vacuum Balzers BF freeze-etching chamber of a FESEM instrument in cryo-mode (HITACHI S-4800, Hitachi, Tokyo, Japan) with secondary electron image resolution of 1.0–1.4 nm at voltages of 1–15 kV. Gels were sublimated for 1 h at 80 °C before image acquisition.

### TEM

Freshly isolated, 4- and 14-day cultured c-kit^+^ cells were recovered from the scaffolds as already described, pelleted and fixed in 3 % glutaraldehyde, and processed for transmission electron microscopy (TEM) as described previously [13]. Cells were visualized using a transmission electron microscope at 60 kV (EM 400 T, Philips, Eindhoven, The Netherlands).

### IHC

At the indicated time points, culture medium was removed and samples fixed in 10 % formalin for at least 24 h at 4 °C. Scaffolds were decalcified using EDTA for 48 h, sectioned, ethanol fixed and embedded in paraffin according to the standard protocols of the Institute of Pathology, University Hospital Aachen. Paraffin sections of 3 μm were deparaffinized, hydrated using decreasing ethanol series and subject to heat-induced antigen retrieval using citrate buffer pH = 6.0. Immunohistochemistry stainings were performed using an Autostainer platform for immunohistochemistry (IHC) (Dako Cytomation). Primary antibodies used were specific for CD31 (1:100, rabbit polyclonal, Abcam), CD45(1:100, rabbit polyclonal, Abcam). Primary antibodies were diluted in Dako antibody diluent and incubated for 1 hour at room temperature. StreptABC complex/HRP followed by DAB for color development was used according to the Dako real detection system instructions (Dako Cytomation, K5001). Reticulin stain and hematoxilin-eosin stains were additionally performed according to routine histology protocols.

### RT-qPCR

Isolation of total RNA from cultures was done with Tripure Isolation Reagent (Roche, Mannheim, Germany) [13, 14]. In brief, a high-capacity cDNA Reverse Transcriptase Kit (Applied Biosystems, Darmstadt, Germany) was used for RNA reverse transcription product amplification that was done using a 7300 Real-Time PCR System and SYBR green (Applied Biosystems, Darmstadt, Germany) running an amplification cycle consisting of 10-min denaturation at 95 °C, additional 40 cycles of denaturation at 95 °C for 15 s, and final extension of 1 min at 60 °C. The housekeeping gene GAPDH was used for normalization of data. Gene expression on collagen I/III gel controls was set to one using the 2^−∆∆ct^ method [8]. Primers used (Eurofins MWG Operon, Ebersberg, Germany) are listed in Additional file 1: Table S1.

### Calcium and phosphate assays

Calcium and phosphate content of culture supernatants was measured at weeks 1, 2, and 3 after cell seeding using a standard autoanalyzer. Alternatively, a commercially available colorimetric-based calcium assay kit (RANDOX, Crumlin, UK) was used to determine the amount of calcium released from the β-TCP scaffolds. The assay was carried out according to the manufacturer’s instructions and absorbance measured at 578 nm using an Infinite® M200 microplate reader (Tecan, Männedorf, Switzerland).

### Mouse transplantations

Six- to eight-week-old C57BL/6 mice were obtained from Jackson Laboratories (Maine, USA). Animals were kept at the Animal Research Facility Children’s Hospital, Harvard Medical School, Boston, USA. Animals were handled according to institutional regulations and transplantations done according to standard protocols (mouse protocol number 13-04-2393R). mBMSCs were expanded in vitro for 1 week and then seeded on 500- and 800-μm β-TCP scaffolds in combination with collagen I/III or Matrigel®. Cell/matrix/scaffold hybrids were transplanted subcutaneously into the C57BL/6 mice.

### Statistical analysis

Data presented was obtained from at least three independent donors. Results express mean ± SD, unless stated otherwise. Statistics as well as graphical representations were performed using GraphPad Prism^TM^ 5.0 (GraphPad Software Inc., San Diego, USA). Statistical significance of data results from one-way ANOVA followed by Tukey’s post hoc test (analysis of three or more groups). Significant differences were considered when *p* < 0.05.

## Results

### β-TCP scaffold characterization

We first characterized the 3D porous β-TCP scaffolds, which were designed to mimic the spongious, structural part of the bone in terms of (i) osteoconductivity (porosity allowing for cell penetration and/or attachment and growth), (ii) osteogenicity (local osteoblastic mineral formation allowing for matrix calcification) [18], (iii) transplantability, (iv) inertness, and (v) hematopoiesis-supporting capacities. As pore size is known to affect cell attachment, proliferation, and migration [19], we tested the biological performance of β-TCP scaffolds with two different pore sizes—500 and 800 μm in diameter—similar to the geometry of the human bone and as used for replacement strategies [20, 21]. We applied scaffolds with 500- and 800-μm pore sizes in order to (a) simulate human bone properties, (b) maximize the surface area and volume available for stromal cell expansion, and (c) support bone ingrowth. As the literature reports strongly variable pore sizes ranging from 300 to 900 μm to be optimal, we analyzed if pore size matters for the recapitulation of extramedullary niches containing two cell types—adhering stromal cells and hematopoietic stem and progenitor cells [22].

**Fig. 1:**
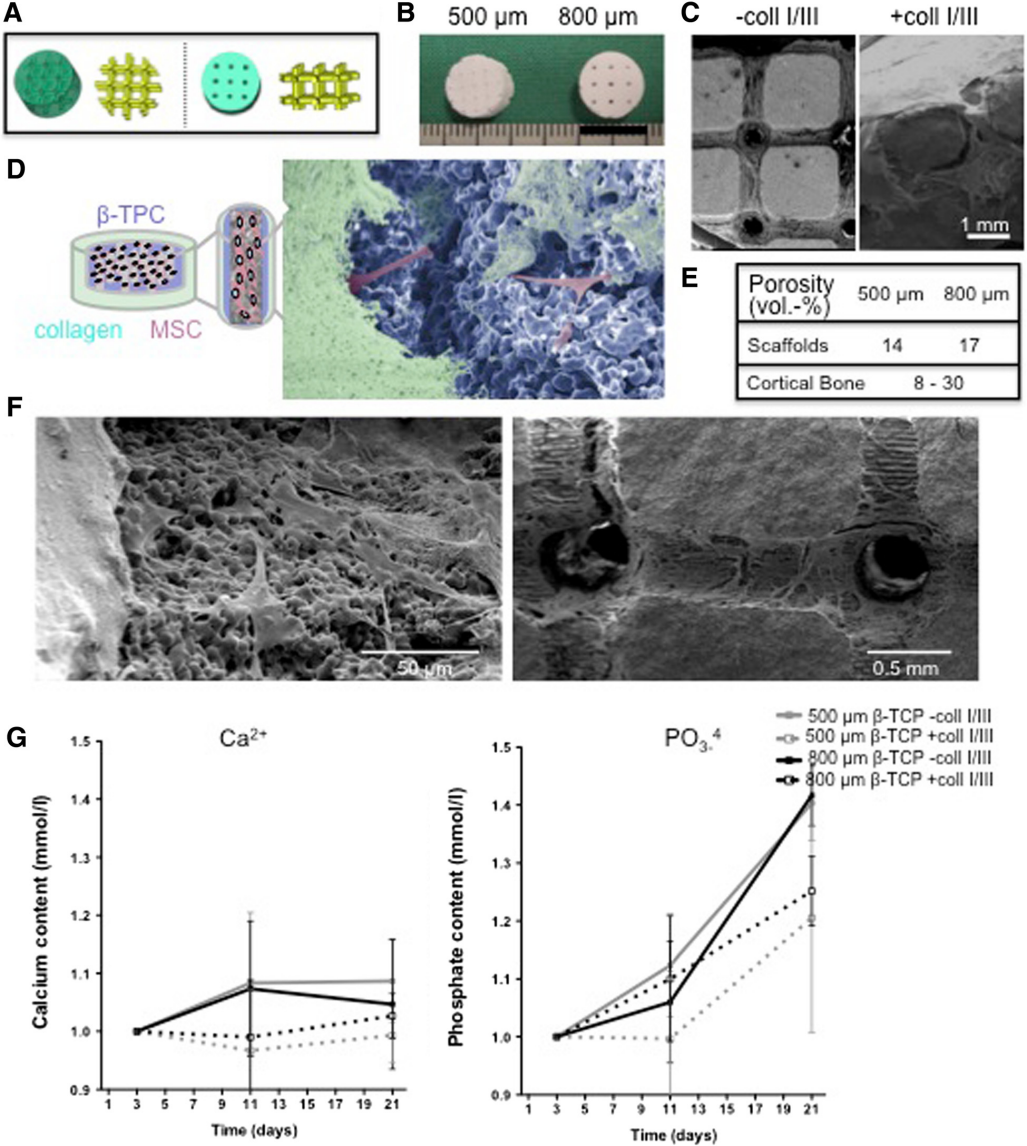
Characterization of β-TCP scaffolds. **a** Negative mold used for wax casting during production of the different β-TCP scaffold geometries. **b** Macroscopic view of the β-TCP scaffolds. **c** SEM view of the β-TCP scaffolds. **d** Schematic illustration of the long-term cultures consisting of three main components: mesenchymal stromal cells (MSCs; *pink*), β-TCP (*blue*), and collagen I/III (*green*). **e** Table of porosites. **f** Cell morphology and cell spreading on scaffolds analyzed by SEM. **g** Release of calcium (Ca^2+^) and phosphate (PO_4_^3−^) to the culture supernatant. Concentrations were measured in the supernatants of β-TCP cultures at days 3, 11, and 21 after the beginning of the culture. Concentrations were normalized to 1 at day 3 and are presented in mmol/L. Results depict mean ± SD of three independent experiments

β-TCP scaffolds were generated with interconnected macropore geometries for better cell growth, migration, and nutrient flow (Fig. 1a–g). The porosity of 500- and 800-μm scaffold was 14 and 17 vol.-%, respectively (Fig. 1e), comparable to the porosity of human cortical bone that is known to go from up to 8 % in young individuals to 24–28 % in elderly individuals [23, 24]. Maximized 500/800-μm scaffold magnitudes were provided for 3D culture including a surface area of 648/584 mm^2^ for stromal cell expansion and a volume of 51/60 mm^3^ for HSPC expansion.

For scaffold characterization experiments, we seeded hMSCs onto 500- and 800-μm β-TCP in the presence or absence of collagen I/III (Fig. 1c, d, f). We first assessed the inertness of β-TCP scaffolds indicated by release of Ca^2+^ and PO_4_^3−^ in culture supernatants after 3, 11, and 21 days of culture (Fig. 1g). Analysis confirmed a minor increase in Ca^2+^ concentration in the culture medium of β-TCP-containing scaffolds, ca. 0.1 mmol/L for 500- or 800-μm β-TCP scaffolds. The PO_4_^3−^ concentration (Fig. 1g) increased over time in all β-TCP-containing supernatants (increase of 0.2–0.4 mmol/L) within ranges that are negligible and cannot be considered as indicators of spontaneous osteogenesis.

### Assessment of the β-TCP scaffold potential for osteogenic differentiation

**Fig. 2:**
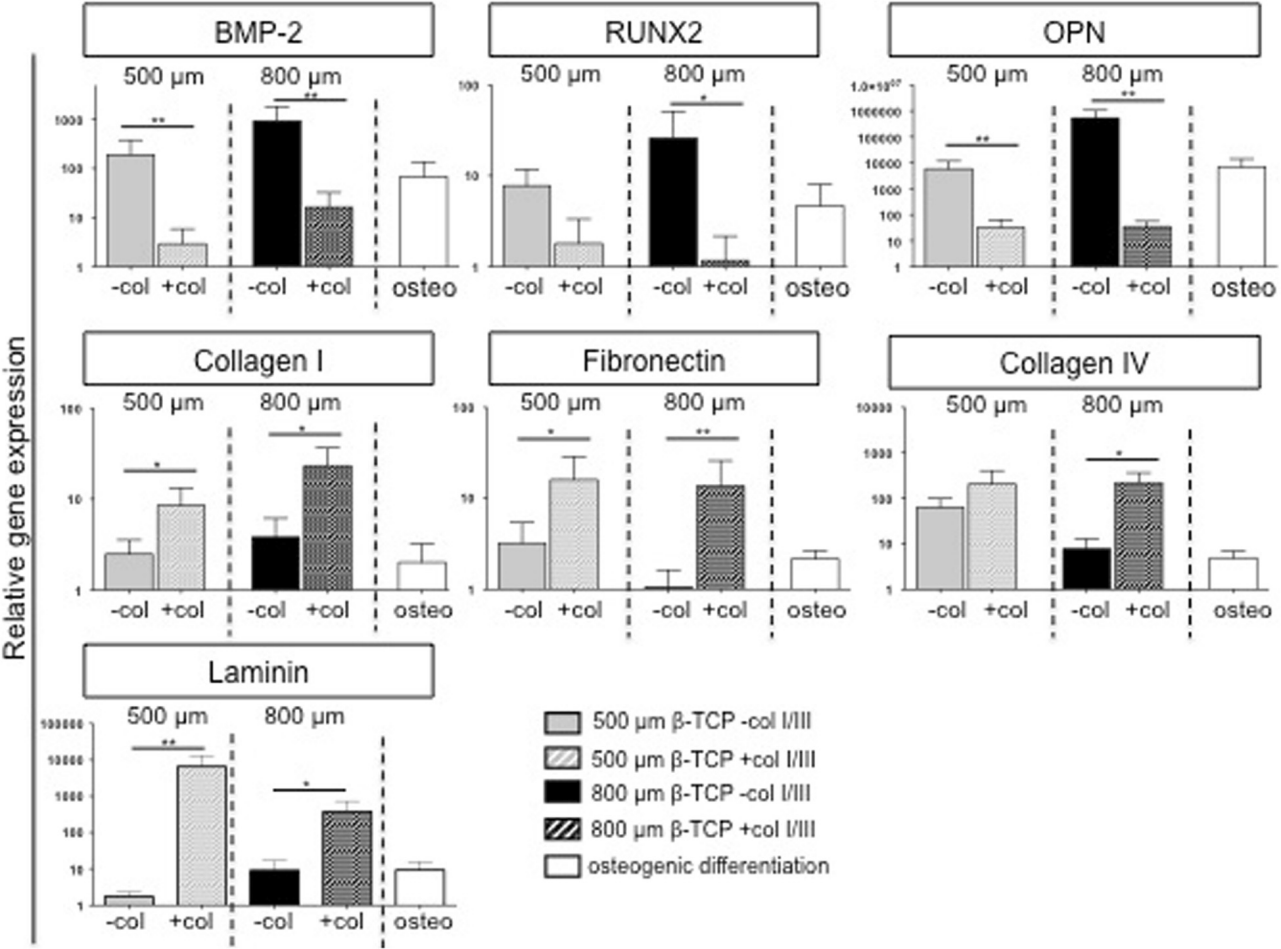
Osteogenic marker and extracellular matrix expression analysis of long-term MSC cultures on β-TCP scaffolds. The osteogenic markers BMP-2, RUNX2, and OPN as well as the extracellular matrix proteins collagen I, fibronectin, collagen IV, and laminin were analyzed after 8 weeks in culture. Data were calibrated relative to MSCs growing in collagen I/III gels, where gene expression was set to 1 for all genes. GAPDH was used as a housekeeping gene for normalization. Gene expression is presented as mean ± SD of three independent experiments and depicted on a logarithmic scale

To determine if β-TCP induces a spontaneous osteogenic differentiation, we next analyzed the effect of β-TCP and collagen I/III on hMSC growth, osteogenic differentiation, and matrix production assessed by gene expression (Fig. 2). β-TCP scaffolds had good osteoconductivity as indicated by hMSC attachment, migration, and survival in the interconnected macropores of β-TCP scaffolds (Fig. 1f; Additional file 2: Figure S1).

To analyze if the scaffold induced a spontaneous osteogenic differentiation of hMSCs, we seeded hMSCs in direct contact to 500/800-μm β-TCP scaffolds (Fig. 2). To dissect the additional effect of an extracellular matrix, we compared the spontaneous differentiation potential side-by-side to β-TCP/collagen I/III scaffolds and looked at hMSCs subjected to standard osteogenic differentiation protocols as a positive control (Fig. 2). BMP-2 and RUNX2 as early markers of osteogenic differentiation as well as the late marker osteopontin were not significantly up-regulated after 3 weeks of culture under nearly all β-TCP conditions, independent of the presence of collagen I/III gels (not shown). After 8 weeks of culture, the induction of early and late markers was maintained for cells cultured in β-TCP scaffolds in the absence of collagen I/III gels and decreased in the presence of collagen I/III gels (Fig. 2). This effect might be due to significant matrix remodeling that occurs in the presence of collagen I/III-containing scaffolds as indicated by the up-regulation of collagen I, fibronectin, laminin, and collagen IV in 8-week cultures (Fig. 2). Taken together, gene expression analysis provides no evidence for spontaneous osteogenesis upon in vitro culture or co-culture of hMSCs.

### Characterization of the β-TCP/matrix hybrids as extramedullary hematopoietic niches in vitro

We aimed to generate transplantable extramedullary hematopoietic niches and next analyzed the effect of β-TCP constructs on murine hematopoiesis in vitro. We combined 500- and 800-μm β-TCP scaffolds with either collagen I/III [8] or Matrigel® to improve cell engraftment and proliferation [25]. mBMSCs were cultured for 1 week on β-TCP scaffolds (with and without collagen I/III) before freshly isolated c-kit^+^ HSPCs from murine BM were added. In order to decide on the optimal combination of extracellular matrix and β-TCP pore size for future transplantation experiments, we assessed (i) viability, (ii) maintenance of a primitive HSPC phenotype and lineage differentiation, (iii) proliferation, and (iv) extracellular matrix remodeling.

**Fig. 3:**
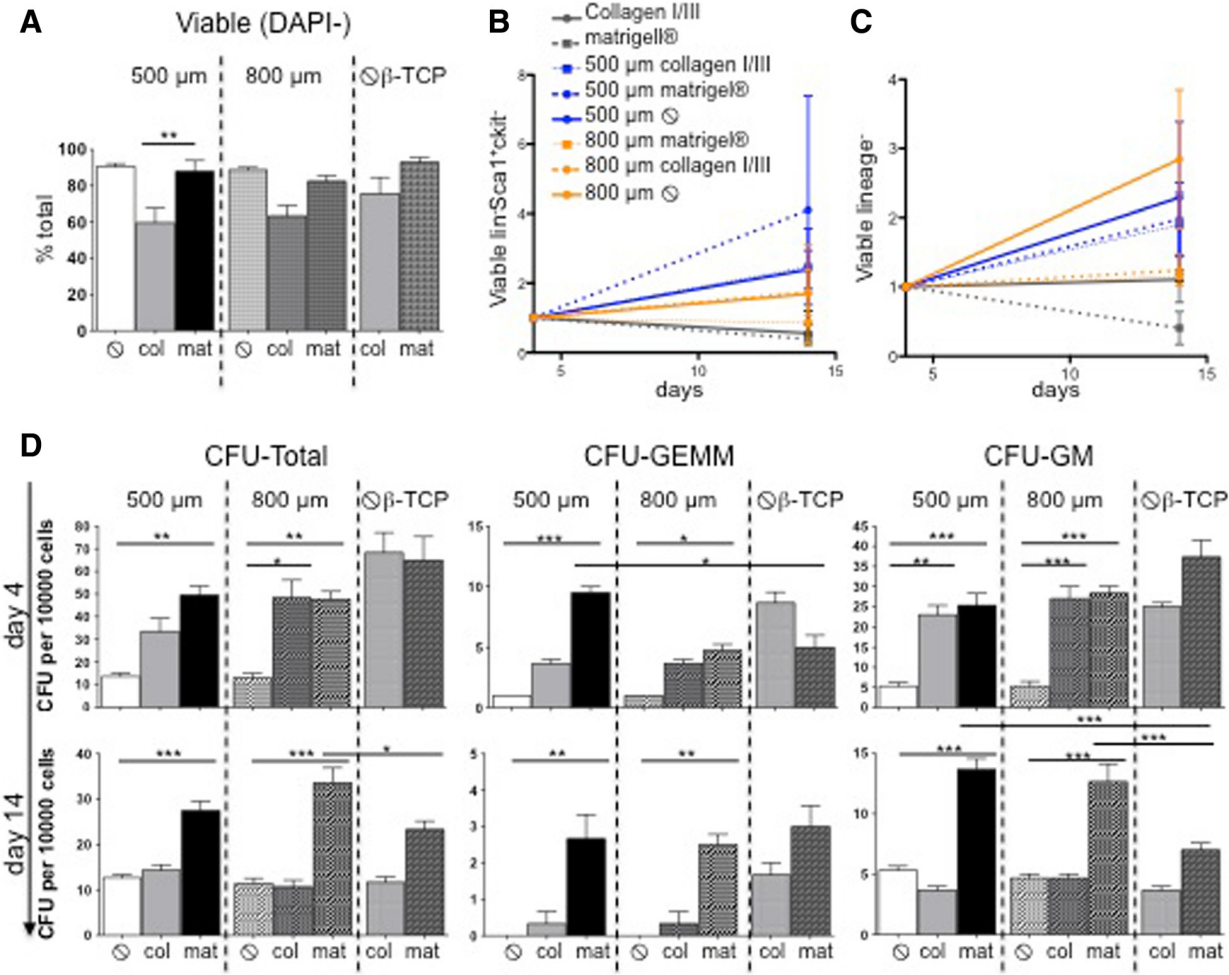
Hematopoietic stem cell characterization in β-TCP scaffolds in vitro. C-kit^+^ cells were subjected to the different β-TCP/matrix hybrids over 14 days in vitro. **a** Percentage of DAPI^−^-viable cells after 14 days of c-kit^+^ cell culture with and without collagen I/III/Matrigel® on β-TCP scaffolds in the presence of MSC support. **b** Expansion/decrease of lin^−^Sca1^+^c-kit^−^ stromal cells normalized to day 4 of the culture. **c** Expansion/decrease of lin^−^ normalized to day 4 of the culture. Mean ± SD of three independent experiments. **d** Colony-forming potential of cells isolated from 500- and 800-μm β-TCP scaffolds with and without collagen I/III/Matrigel® in the presence of MSC support at day 4 and day 14. Results are mean ± SD of three independent experiments. *CFU* colony-forming unit, *GEMM* granulocyte-erythrocyte-macrophage-megakaryocyte, *GM* granulocyte-monocyte.  without

Maintenance of the primitive hematopoietic stem and progenitor cell phenotype was monitored after 5 and 12 days for the human setting (Additional file 3: Figure S2A) and 4, 7, and 14 days for the murine setting (Fig. 3a–c; Additional files 4 and 5: Figures S3–S4). Murine hematopoietic stem cells can be characterized in the bone marrow by being lineage-marker negative, c-kit^+^ and Sca1^+^ (LSK population; lin^−^Sca1^+^c-kit^+^), while hematopoietic progenitor cells are lineage-marker negative, Sca1^−^ and c-kit^+^ (LK population; lin^−^Sca1^−^c-kit^+^). After 14 days, the viability in the β-TCP scaffolds alone or in combination with Matrigel® was independent of the pore size (Fig. 3a). The presence of a collagen matrix reduced the cell viability, probably due to significant matrix remodeling, as discussed before. The stromal cells in the collagen scaffolds can be characterized as lin^−^Sca1^+^c-kit^−^. We quantified the percentage of lin^−^Sca1^+^c-kit^−^ stromal cells at day 4 and monitored their increase/decrease over time (Fig. 3b, c). In particular, 500-μm scaffolds had a positive effect on stromal cell expansion. We next assessed the maintenance of the LSK and LK phenotypes in vitro. Our results showed that β-TCP/extracellular matrix hybrids more robustly maintained a phenotypical LSK fraction over time compared to β-TCP alone (Additional file 4: Figure S3). C-kit^+^ expression was shown to decrease over time in vitro independent of the hematopoietic stem and progenitor phenotype [26]. We thus quantified lineage-negative cells in the scaffolds over time as this population is enriched in hematopoietic stem and progenitor cells. The presence of 500-μm scaffolds showed a relative expansion of lineage-negative cells while this population maintained stable in the other conditions. We confirmed these results by using human HSPCs. In humans, the subset of CD34^+^CD38^−^ HSPCs corresponds to a more primitive phenotype. Our data in short-term co-cultures showed that CD34^+^CD38^−^ HSPCs were maintained most sufficiently in β-TCP scaffold containing collagen I/III (Additional file 3: Figure S2A), comparable to our results in the murine system.

In order to evaluate not only the HSPC phenotype but also their function among the different scaffold combinations as the ultimate readout for the maintenance of hematopoietic stem cell capacities, we performed colony-forming assays at day 4 and day 14 (Fig. 3d). Our data showed that murine cells cultured on β-TCP scaffolds alone for 4 days had significantly impaired CFU potential and HSPC function compared to all the other conditions. The presence of Matrigel® alone resulted in the significantly highest total number of colonies, and the combination of β-TCP/Matrigel® led to the highest number of CFU-GEMM colonies, representing multipotent myeloid progenitor cells. As expected, the CFU potential globally decreased after 14 days, but HSPCs cultured in the combination of β-TCP/Matrigel® scaffolds (*p* < 0.001 compared to 500-μm β-TCP and *p* < 0.002 compared to 800-μm β-TCP) gave rise to the significantly highest number of colonies, in particular to primitive CFU-GEMM colonies, indicating that the function of HSPCs was maintained under these conditions.

**Fig. 4:**
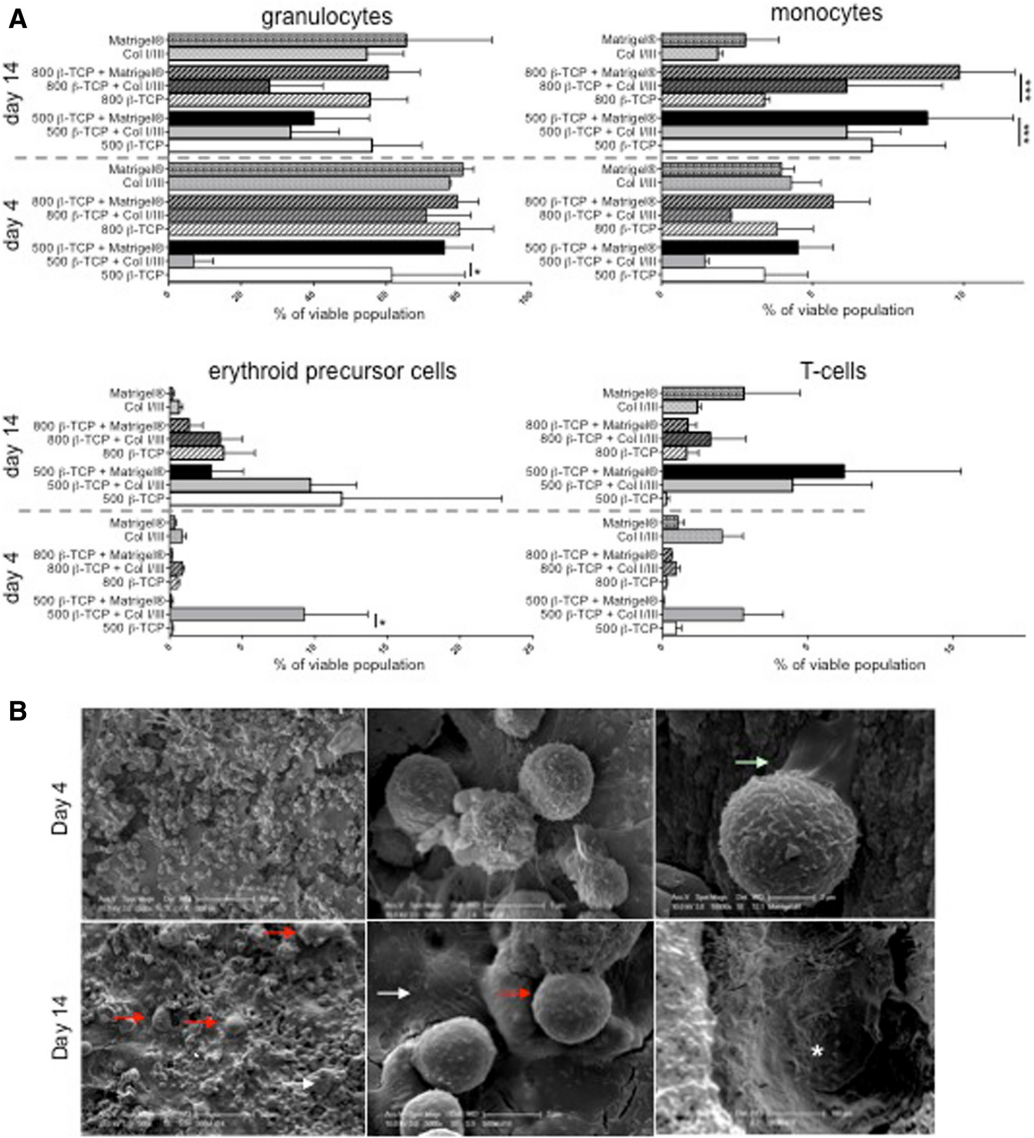
Analysis of trilineage differentiation of c-kit^+^ HSPCs on β-TCP scaffolds in vitro. **a** Differentiation potential of c-kit^+^ cells cultured for 4 and 14 days on β-TCP scaffolds with and without collagen I/III gel/Matrigel® in the presence of MSC support: granulocytes (Gr1^+^CD11b^+^), monocytes (Gr1^−^CD11b), erythroid precursor cells (Gr1^−^CD11b^−^CD3^−^CD19^−^), and T cells (Gr1^−^CD11b^−^CD3^+^). Percentage of viable cells is shown as mean ± SD of three independent experiments. **b** SEM of c-kit^+^ cells cultured on 500-μm β-TCP/Matrigel® scaffolds. *Very left column* highlights cell spreading and detailed cell morphology of day 4 and day 14 cultures. C-kit^+^-derived cells (*red arrows*) are in close contact to MSCs (*white arrows*). Cell extensions at day 4 in detail (*green arrow*). View from the interior of the β-TCP macropores at day 14 (*asterisk*)

We next analyzed the lineage differentiation potential and asked whether or not scaffold combinations induce a lineage bias (Fig. 4a; Additional file 5: Figure S4). We analyzed granulocytes (Gr1^+^CD11b^+^), monocytes (Gr1^−^CD11b^+^), B-cells (Gr1^−^CD11b^−^CD19^+^), T cells (Gr1^−^CD11b^−^CD3^+^), and erythroid cells (Gr1^−^CD11b^−^CD3^−^CD19^−^) in the murine in vitro culture system at day 4 and day 14. All culture conditions induced myeloid differentiation into granulocytes and monocytes. The frequency of granulocytes decreased in tendency over the culture of 14 days, while the frequency of monocytes increased. After 14 days, the presence of Matrigel® in 500 and 800 μm significantly stimulated monocyte differentiation as compared to β-TCP scaffolds alone. We also detected both lymphoid (mainly T cells) and erythroid differentiation in all culture conditions, indicating that the scaffold-matrix hybrids support both trilineage differentiation as well as maintenance of primitive, functional HSPCs.

Next, we dissected the spatial distribution of human CD34^+^ HSPCs and murine c-kit^+^ cells in β-TCP/matrix hybrids combinations (Fig. 4b; Additional file 3: Figure S2B; Additional files 6, 7, and 8: Figures S5–S7). HSPCs growing on β-TCP/Matrigel® were spread on top of the scaffolds as single cells or in close proximity to mBMSCs (Fig. 4b) or growing inside scaffold pores attached to the Matrigel® surface (Fig. 4b; Additional file 6: Figure S5). After 14 days, the cell density was significantly increased but cells were predominantly positioned on top of the scaffolds/Matrigel, indicating that Matrigel does not support the migration of HSPCs due to high density and overall low porosity (Additional file 6: Figure S5). This hypothesis was supported by imaging using SEM in cryo-mode (Additional file 6: Figure S5ii–iii). The Matrigel® porosity—as a result of the gelation process—was higher at the top part of the gel and lower at the bottom, a limitation which eventually led to the rare cell migration into the Matrigel®. Collagen I/III gels showed high cell densities on top as well as significant migration of hematopoietic cells inside the gel and cells growing in close proximity to mBMSCs and collagen fibers (Additional file 7: Figure S6). Similarly, the addition of β-TCP to collagen I/III gels supported cell proliferation as well as migration both in the murine and human settings (Additional file 3: Figure S2B; Additional file 8: Figure S7A, B). In β-TCP scaffolds, the cell proliferation and density appeared significantly impaired despite the presence of a robust mBMSC layer covering the β-TCP surfaces (Additional file 8: Figure S7A, B). This leads to the suggestion that β-TCP scaffolds are supportive of the growth of mBMSCs but that hematopoietic cells require an additional matrix structure.

### Characterization of the β-TCP/matrix hybrids as extramedullary hematopoietic niches in vivo

**Fig. 5:**
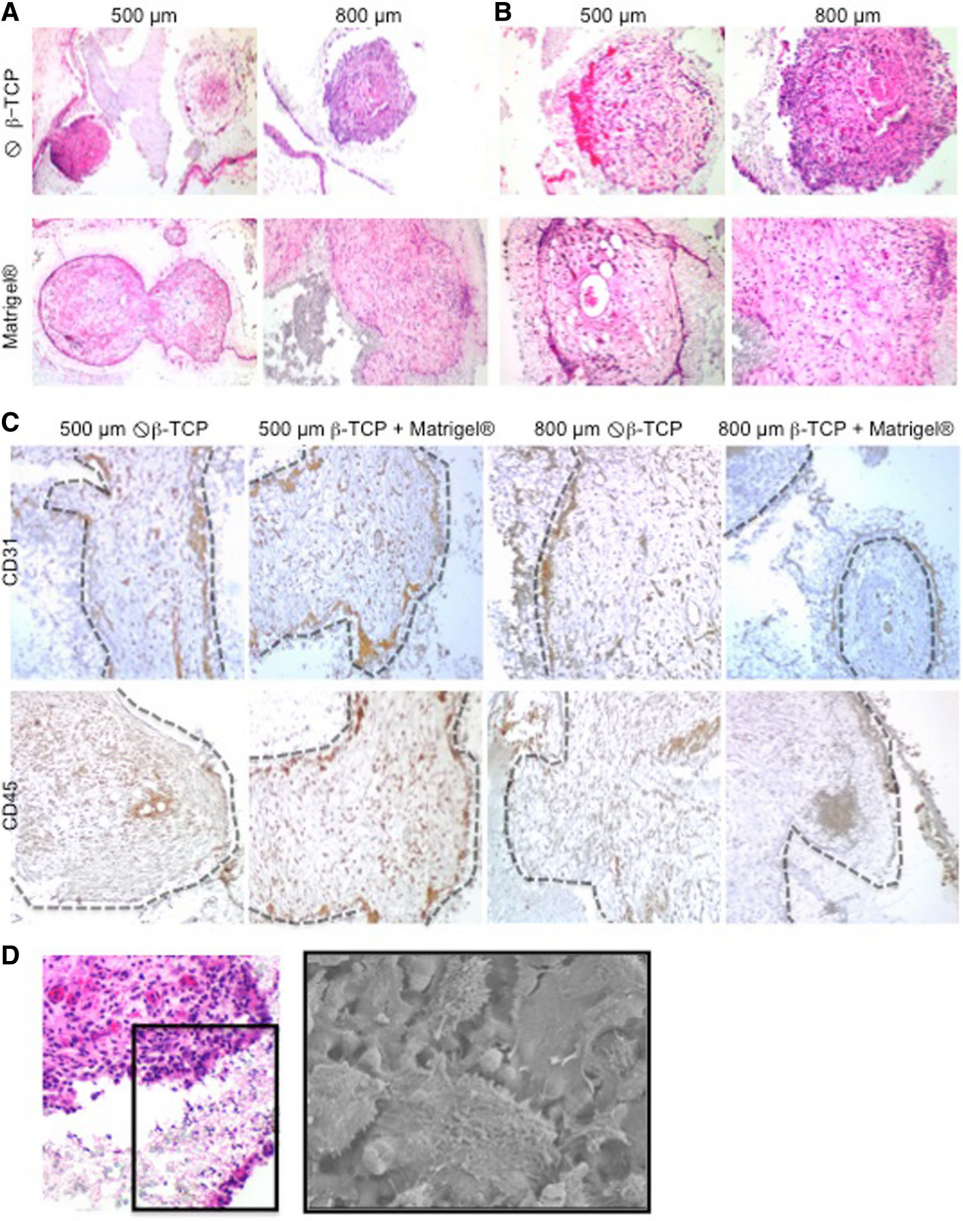
Histomorphology. 500μm or 800 μm β-TCP scaffolds were pre-seeded with mBMSC in the presence or absence of Matrigel^®^. Scaffolds were transplanted subcutaneously into C57B1/6 mice and analyzed for hematopoietic cell recruitment to sites of extramedullar hematopoiesis and hematopoietic tissue formation after 4 and 8 weeks. **a** Hematoxilin-eosin (HE) staining of the explanted scaffolds 8 weeks after transplantations. Magnification 100x. **b** Higher magnification of the explanted scaffolds after 8 weeks. Magnification 200x. **c** Immunohistochemistry for the endothelial surface marker CD31 and the pan-hematopoietic marker CD45 in explanted scaffolds 8 weeks after transplantation. **d** HE staining and by SEM for the identification of osteoclasts at the border of the β-TCP scaffold macropores in explants 4 weeks after transplantation

**Fig. 6:**
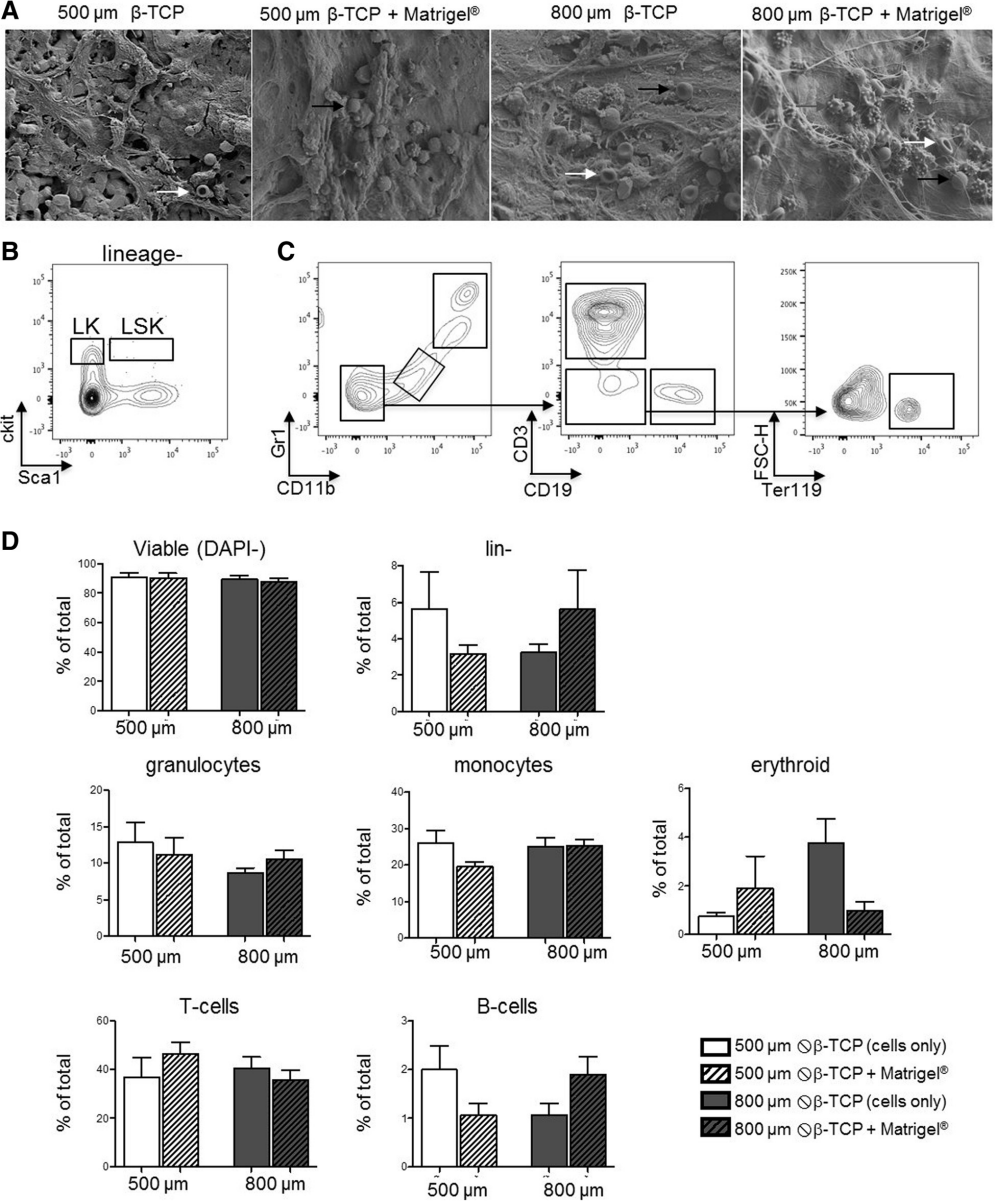
**a** SEM imaging of the explanted scaffolds 4 weeks after transplantation. *White arrows*: erythrocytes; *black arrows*: T cells; *red arrows*: thrombocytes; *gray arrow*: neutrophils/granulocytes; *asterisk*: β-TCP. **b-c** Gating strategy for the flow cytometry: Hematopoietic stem (LSK) and progenitor cells (LK) were identified by being negative for lineage markers and by the expression of Sca1 and c-kit. Lineages were identified by the following stainings: granulocytes (Gr1^+^CD11b^+^), monocytes (Gr1^−^CD11b^−^), erythroid precursor cells (Gr1^−^CD11b^−^CD3^−^CD19^−^), T cells (Gr1^−^CD11b^−^CD3^+^), B cells (Gr1^−^CD11b^−^CD19^+^). **d** Flow cytometry analysis of the explanted scaffolds 4 weeks after transplantations. Surface marker expression is shown as a percentage of the viable cell population; mean ± SD of three independent experiments

To test the capacity of β-TCP scaffolds for supporting recruitment of hematopoietic cells and extramedullary hematopoiesis, 500- and 800-μm β-TCP scaffolds pre-seeded with mBMSCs were transplanted subcutaneously into C57BL/6 mice in the presence and absence of collagen I/III or Matrigel®. Hematopoiesis in the explanted scaffolds was evaluated 4 and 8 weeks after transplantation by flow cytometry, immunohistochemistry, and SEM (Figs. 5 and 6).

We performed H&E staining (Fig. 5a, b) in order to analyze the tissue and cell organization within the scaffolds. Histomorphological overviews in low magnification demonstrated that all scaffold pores—both in the periphery and in the central areas—were composed of a strongly vascularized extracellular matrix, partially adipose tissue, prominent histocyte-like cells aligning the β-TCP surface, and accumulations of hematopoietic cells in close proximity to vessels and the β-TCP surface (Fig. 5a). Higher magnification demonstrated dense clusters of hematopoietic cells in particular at the scaffold/matrix interface (Fig. 5b).

CD31 immunohistochemistry confirmed a dense vascular network in the scaffold, an important requisite for successful long-term engraft of scaffolds and recruitment of hematopoietic cells (Fig. 5c). CD31-positive cells were also seen at the β-TCP/tissue interface. CD45 staining highlighted the spatial organization of hematopoietic cells in dense clusters in proximity to both the vasculature and the scaffold interfaces (Fig. 5c). As observed in vitro, the β-TCP surface seeded with mBMSCs appears to provide appropriate conditions for the development of a bone-like niche, as we also noted the presence of multinucleated osteoclasts and osteoblasts. The presence of osteoclasts was confirmed by SEM showing spindle-shaped cells with brush-like microvilli (Fig. 5d).

We next sought to characterize the identity of hematopoietic cells (Fig. 6a–d). All β-TCP/matrix hybrid combinations were able to recruit hematopoietic cells to the site of transplantation as SEM confirmed hematopoietic cells inside the pores of the transplanted scaffolds (Fig. 6a).

Our first question was whether the implanted scaffolds promote extramedullary hematopoiesis including the presence of hematopoietic stem and progenitor cells. Flow cytometry confirmed the presence of cells of all hematopoietic lineages: myeloid (CD11b^+^Gr1^+^), lymphoid (Gr1^−^CD11b^−^CD19^+^ or CD3^+^), and erythroid (Gr1^−^CD11b^−^CD19^−^CD3^−^ Ter119^+^) lineages (Fig. 6c, d). We did not detect an HSC-enriched LSK population but hematopoietic progenitor cells as indicated by the presence of lineage-negative and also myeloid progenitor cells (LK population). These data indicate that the majority of hematopoietic cells were recruited to the site of transplantation and do not represent in situ, extramedullary hematopoiesis. We did not observe significant differences in the recruitment of cell lineages in the different culture conditions, demonstrating that all conditions are suitable for ectopic transplantation independently of β-TCP pore size or presence of extracellular matrix.

## Discussion

The BM microenvironment is not transplantable making it challenging to dissect the regulatory mechanisms within the BM niche. We envisioned a widely available, easy reproducible, simple, and well-defined system for the creation of a transplantable human BM microenvironment. Establishing such a system would include not only a detailed characterization of the hematopoietic and mesenchymal fractions upon culture/transplantation but also the design of an appropriate scaffold material and structure. For the co-culture of HSPCs and mBMSCs/hMSCs, we applied β-TCP-printed scaffolds containing macro- and micropores similar to the bone—of inorganic (hydroxyapatite mineral) composition—in combination with an extracellular matrix gel—of organic (collagen type I matrix) composition.

As β-TCP materials are unstable when exposed to body fluids due to their small granule size, porosity, and higher dissolution rate compared to other types of bioceramics [27], we first set up to analyze the effects of β-TCP dissolution on the cultured cells. Typically high phosphate levels induce osteogenic differentiation, so we asked whether or not β-TCP scaffolds in our setting induced spontaneous osteogenic differentiation of MSCs by Ca/P release to the culture media. Our analysis revealed negligible Ca/P release and the exclusion of the possibility for spontaneous MSC-derived osteogenesis that could be detrimental for hematopoietic production. Importantly, we showed that our β-TCP scaffolds have the ability to support MSC growth and extracellular matrix production in cultures up to 8 weeks without limiting effects.

Our in vitro data suggest that complete hematopoiesis is promoted by β-TCP scaffolds in combination with structural proteins and cytokines provided by Matrigel®. First, β-TCP/Matrigel® scaffolds more robustly maintained a primitive LSK fraction over time, with the Matrigel® component being responsible for maintenance of HSPCs within the scaffolds. Second, hematopoietic differentiation in both myeloid and lymphoid lineages was supported after 14 days in Matrigel®-containing conditions. Third, the CFU potential of β-TCP/Matrigel® scaffolds was significantly increased compared to other conditions implicating that Matrigel supports both the functional maintenance of HSPCs and their differentiation capacity. Fourth, β-TCP/Matrigel® scaffolds supported the remodeling of the ECM as an important requisite for cell differentiation and growth.

In vivo, β-TCP/Matrigel® scaffolds resulted in strong hematopoietic recruitment to the sites of the ectopic transplantations as confirmed by the detection of myeloid and lymphoid fractions 4 weeks after transplantations at levels comparable to the relative composition of the BM. Multinucleated osteoclasts and typically spindle-shaped osteoblasts were seen at the β-TCP scaffold/ECM interface. CD31-positive layers of recruited endothelial cells and adipose cells, typical for vascular niches, were both identified in explants showing the most efficient collagen matrix deposition. Adipose cells were regarded as simple as BM fillers but are now known to be negative regulators of the hematopoietic microenvironment [28]. BM tissue formation in our setting was mainly promoted by Matrigel®, a biodegradable basement membrane protein extracted from Engelbreth-Holm-Swarm sarcoma mouse cells, typically used to recreate 3D environments stimulating tissue formation. The main components of Matrigel® are known to be structural proteins laminin, collagen IV, and enactin, but numerous other intracellular proteins are present. Around 1300 proteins (either cytoplasmatic or nuclear) were identified in Matrigel® composition, and among those were collagen IV; actin; spectrin; tubulin; dynactin; filamin structural proteins like fibronectin, dynein, desmin, myosin, transferrin; and intracellular proteins such as adenylate kinase and heat shock family members [29]. Additionally, specific growth and transcription factors such as kruppel-like factor 6, kruppel-like factor 15, and connective tissue growth factor were identified. Future studies will be necessary to dissect which of these factors are most important to support both hematopoiesis and osteogenesis to generate more defined environments.

Our study demonstrates that varying pore sizes of the β-TCP scaffolds from 500 to 800 μm was irrelevant in terms of hematopoietic support function. This is an interesting finding as particle geometry, porosity, pore size distribution, and scaffold continuity are properties known to determine cell/scaffold interactions and thus cell fate [20, 30, 31]. Pore size, however, has been only considered crucial within three main pore size intervals. According to Petite et al., pore sizes smaller than 15–50 μm result in fibrovascular growth, medium-sized pores of 50–150 μm encourage osteogenesis, while pores larger than 150 μm support the ingrowth of mineralized bone [20]. Contradicting studies even report that bone ingrowth is optimal for pore sizes varying from 300 to 900 μm [22].

The β-TCP scaffolds applied in our study had an interconnected net-like structure in the absence of dead-end pockets, macropores with mean pore size 500 or 800 μm, and micropores smaller than 15 μm. Our scaffolds were designed to maximize the surface area for stromal cell expansion, and we were able to demonstrate that these characteristics successfully promote vascular ingrowth as well as promotion of hematopoiesis.

Our engineered bone marrow has the potential to be applied for drug discovery studies and leukemia therapy as well as stem cell transplantation research [32, 33].

## Conclusions

We introduce a multicomponent system that is optimal for the study of hematopoietic-mesenchymal interactions and the recapitulation of the native BM microenvironment in a transplantable mouse model. Macro- and microporous β-TCP scaffolds in combination with a Matrigel®-based component optimally supported hematopoiesis, including hematopoietic recruitment, proliferation, and differentiation as well as ECM remodeling. β-TCP/Matrigel® scaffolds further promoted vascularization and allowed for mature bone deposition and hematopoiesis—stroma interactions in subcutaneous ectopic transplantations of the BM niche in a murine model.

## Additional files

Additional file 1: Table S1. Primers used for RT-qPCR. Additional file 2: Figure S1. Representative SEM images of 500- or 800-μm β-TCP scaffolds pre-seeded with hMSCs maintained in culture for 3 weeks with or without addition of collagen I/III. Collagen I/III embedded with hMSCs cultured under standard conditions were undifferentiated controls. Collagen I/III gels with embedded hMSCs cultured under osteogenic differentiation conditions were osteogenic controls (xii). Additional file 3: Figure S2. (A) Monitorization of the CD34^+^CD38^−^ primitive phenotype in human-derived CD34^+^ progenitors co-cultured with hMSCs for 5 and 12 days in the different β-TCP/matrix hybrids. On the left data presented is a mean ± SD of three independent experiments; on the right, dot plots of one representative experiment are shown. (B) Representative SEM images CD34^+^ HSPCs co-cultured for 12 days in 800-μm β-TCP scaffolds in the presence of hMSC-containing collagen I/III matrix. CD34^+^ HSPCs (red arrows) are seen in close contact to hMSCs (white arrows) within the scaffold macropores. β-TCP scaffolds (asterisks) were reinforced with collagen I/III (green arrows). Additional file 4: Figure S3. Dot plot representations of the putative LSK (lin^−^Sca1^+^c-kit^+^) population in starting cultures and 4-, 7-, and 14-day cultures of c-kit^+^-isolated cells in co-culture with mBMSCs on 500- and 800-μm β-TCP scaffolds with or without collagen I/III gels or Matrigel®. Flow cytometry data shown is of one representative experiment. Additional file 5: Figure S4. (A) Representative TEM images of c-kit^+^ cells used for initiating co-cultures after immunomagnetic bead selection. (B) TEM images of 4- and 14-day cultured c-kit^+^ cells on 500-μm β-TCP scaffolds, 500-μm β-TCP/collagen I/III scaffolds, and 500-μm β-TCP/Matrigel® scaffolds. Collagen I/III gels and Matrigel® alone are shown as controls. C-kit^+^ progenitor cell morphology (of freshly isolated cells) consisting of visible scattered chromatin, prominent nucleoli, and approximated cell diameter of 8–10 μm is shown as reference. Additional file 6: Figure S5. Representative SEM images of Matrigel® scaffolds pre-seeded with mBMSCs and later seeded with c-kit^+^-isolated cells; co-cultures were maintained for 14 days (i, iv, v, vii). Detailed morphology of c-kit^+^-derived cells that had migrated inside the Matrigel® after 4 days in culture are also shown (vi, viii, ix). SEM in cryogenic mode (ii) and dry mode (iii) was done to analyze in detail the raw (empty) structure of Matrigel®. Additional file 7: Figure S6. Representative SEM images of collagen I/III gels pre-seeded with mBMSCs and later seeded with c-kit^+^-isolated cells. Co-cultures observed were maintained for 4 days (vi) and 14 days (i, iv, v). SEM in cryogenic mode (ii) and dry mode (iii) shows the typically high microporosity rate of collagen I/III. Additional file 8: Figure S7. (A) Representative SEM images of 800-μm β-TCP scaffolds pre-seeded with mBMSCs and seeded with c-kit^+^-isolated cells. Images of 4-day cultures (i-iii) and 14-day cultures (iv-vi) are shown. (B) All conditions similar to (A) except 800-μm β-TCP/collagen I/III scaffolds were used.
