# Supplementary material for: An engineered multicomponent bone marrow niche for the recapitulation of hematopoiesis at ectopic transplantation sites
Source: J Hematol Oncol. 2016 Jan 25;9:4. doi: 10.1186/s13045-016-0234-9 (PMC4727380; doi:10.1186/s13045-016-0234-9)
Supplement: Additional file 1: Table S1. — Primers used for RT-qPCR. [file 13045_2016_234_MOESM1_ESM.doc]

**Additional file 1: Table S1.** Primers used for RT-qPCR

| **Gene** | **Sequence** |
| --- | --- |
| GAPDH | Fw 5´- GAAGGTGAAGGTCGGAGTCA -3´  Rv 5´- AATGAAGGGGTCATTGATGG -3´ |
| COL1 (Collagen type I) | Fw 5´-TGACCTCAAGATGTGCCACT-3´  Rv 5´-ACCAGACATGCCTCTTGTCC-3´ |
| COL2A (Collagen type IIa) | Fw 5´-TTTCCCAGGTCAAGATGGTC-3´  Rv 5´-CTTCAGCACCTGTCTCACCA-3´ |
| COL4 (Collagen type IV) | Fw 5´-GGATCGGCTACTCTTTCGTGATG-3´  Rv 5´-AAGCGTTTGCGTAGTAATTGCA-3´ |
| FN1 (Fibronectin) | Fw 5´-TGACCTTTTCTGGCTCGTCT-3´  Rv 3´-GTTCAGCACAAAGGGCTCTC-3´ |
| BMP2 (Bone morphogenetic protein 2) | Fw 5‘-ACAAACAGCGGAAACGCCT-3‘  Rv 5‘-CCACCCCACGTCACTGAAG-3‘ |
| RUNX2 (Runt-related transcription factor 2) | Fw 5‘-GCAAGGTTCAACGATCTGAGATT-3‘  Rv 5‘-AAGACGGTTATGGTCAAGGTGAAA-3‘ |
| OPN (Osteopontin) | Fw 5´-AATTGCAGTGATTTGCTTTTGC-3´  Rv 5´-CAGAACTTCCAGAATCAGCCTGTT-3´ |
| PPARG (Peroxisome proliferator-activated receptor gamma) | Fw 5´-TGAAGCTGAACCACCCTGAGT-3´  Rv 5´-CGTGCACAAGGCACTGTTAG-3´ |
| LAMA1 (Laminin I) | Fw 5’-TGACCTTTTCTGGCTCGTCT-3´  Rv 5’-GTTCAGCACAAAGGGCTCTC-3´ |
