# Supplementary figures and images for: An engineered multicomponent bone marrow niche for the recapitulation of hematopoiesis at ectopic transplantation sites
Source: J Hematol Oncol. 2016 Jan 25;9:4. doi: 10.1186/s13045-016-0234-9 (PMC4727380; doi:10.1186/s13045-016-0234-9)

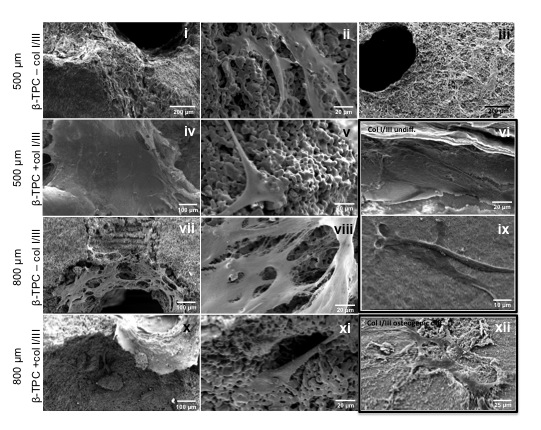

Supplement: Additional file 2: Figure S1. — Representative SEM images of 500- or 800-μm β-TCP scaffolds pre-seeded with hMSCs maintained in culture for 3 weeks with or without addition of collagen I/III. Collagen I/III embedded with hMSCs cultured under standard conditions were undifferentiated controls. Collagen I/III gels with embedded hMSCs cultured under osteogenic differentiation conditions were osteogenic controls (xii). [file 13045_2016_234_MOESM2_ESM.jpg]

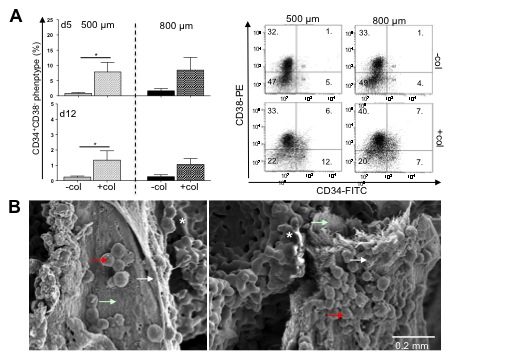

Supplement: Additional file 3: Figure S2. — (A) Monitorization of the CD34+CD38− primitive phenotype in human-derived CD34+ progenitors co-cultured with hMSCs for 5 and 12 days in the different β-TCP/matrix hybrids. On the left data presented is a mean ± SD of three independent experiments; on the right, dot plots of one representative experiment are shown. (B) Representative SEM images CD34+ HSPCs co-cultured for 12 days in 800-μm β-TCP scaffolds in the presence of hMSC-containing collagen I/III matrix. CD34+ HSPCs (red arrows) are seen in close contact to hMSCs (white arrows) within the scaffold macropores. β-TCP scaffolds (asterisks) were reinforced with collagen I/III (green arrows). [file 13045_2016_234_MOESM3_ESM.jpg]

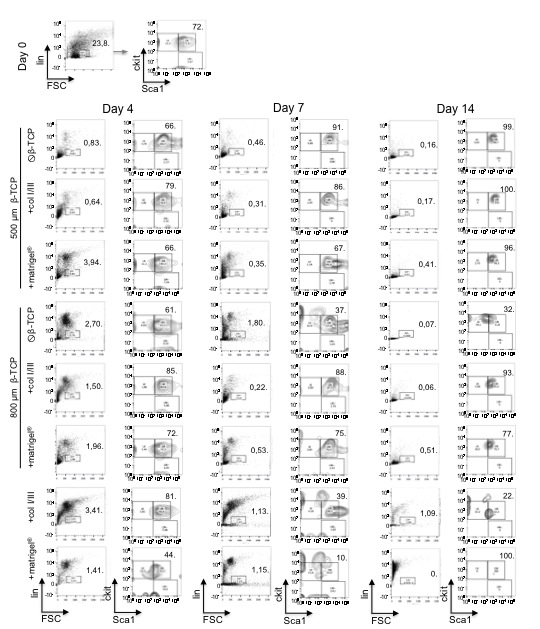

Supplement: Additional file 4: Figure S3. — Dot plot representations of the putative LSK (lin−Sca1+c-kit+) population in starting cultures and 4-, 7-, and 14-day cultures of c-kit+-isolated cells in co-culture with mBMSCs on 500- and 800-μm β-TCP scaffolds with or without collagen I/III gels or Matrigel®. Flow cytometry data shown is of one representative experiment. [file 13045_2016_234_MOESM4_ESM.jpg]

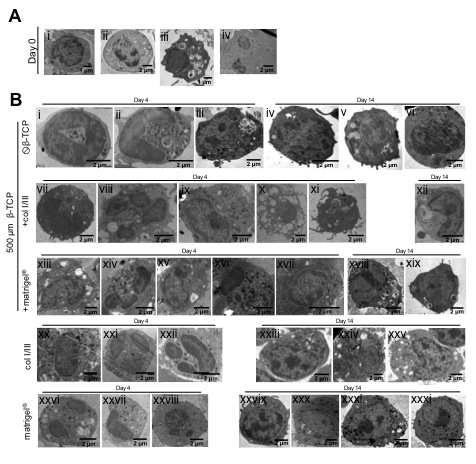

Supplement: Additional file 5: Figure S4. — (A) Representative TEM images of c-kit+ cells used for initiating co-cultures after immunomagnetic bead selection. (B) TEM images of 4- and 14-day cultured c-kit+ cells on 500-μm β-TCP scaffolds, 500-μm β-TCP/collagen I/III scaffolds, and 500-μm β-TCP/Matrigel® scaffolds. Collagen I/III gels and Matrigel® alone are shown as controls. C-kit+ progenitor cell morphology (of freshly isolated cells) consisting of visible scattered chromatin, prominent nucleoli, and approximated cell diameter of 8–10 μm is shown as reference. [file 13045_2016_234_MOESM5_ESM.jpg]

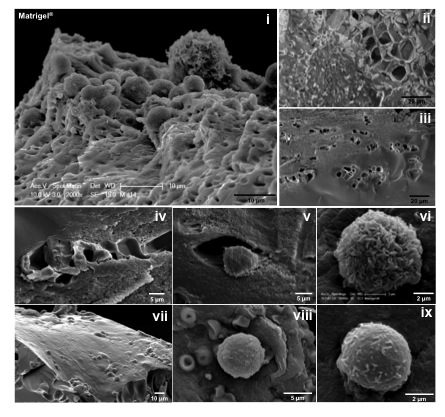

Supplement: Additional file 6: Figure S5. — Representative SEM images of Matrigel® scaffolds pre-seeded with mBMSCs and later seeded with c-kit+-isolated cells; co-cultures were maintained for 14 days (i, iv, v, vii). Detailed morphology of c-kit+-derived cells that had migrated inside the Matrigel® after 4 days in culture are also shown (vi, viii, ix). SEM in cryogenic mode (ii) and dry mode (iii) was done to analyze in detail the raw (empty) structure of Matrigel®. [file 13045_2016_234_MOESM6_ESM.jpg]

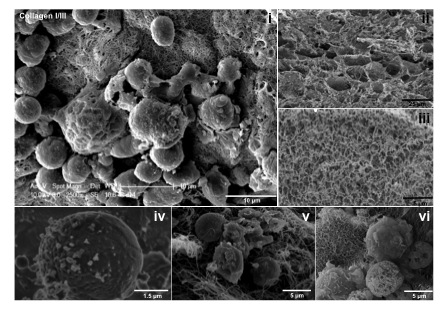

Supplement: Additional file 7: Figure S6. — Representative SEM images of collagen I/III gels pre-seeded with mBMSCs and later seeded with c-kit+-isolated cells. Co-cultures observed were maintained for 4 days (vi) and 14 days (i, iv, v). SEM in cryogenic mode (ii) and dry mode (iii) shows the typically high microporosity rate of collagen I/III. [file 13045_2016_234_MOESM7_ESM.jpg]

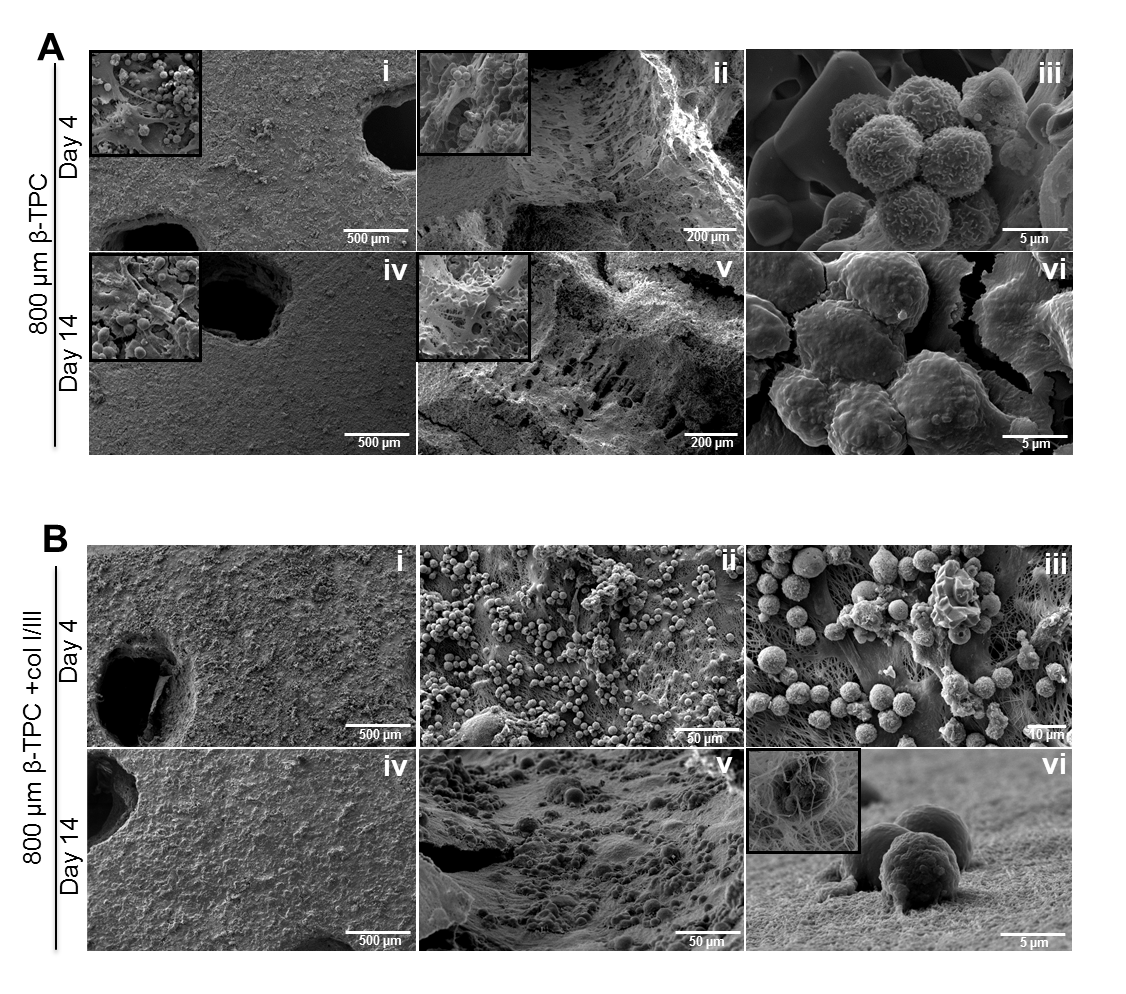

Supplement: Additional file 8: Figure S7. — (A) Representative SEM images of 800-μm β-TCP scaffolds pre-seeded with mBMSCs and seeded with c-kit+-isolated cells. Images of 4-day cultures (i-iii) and 14-day cultures (iv-vi) are shown. (B) All conditions similar to (A) except 800-μm β-TCP/collagen I/III scaffolds were used. [file 13045_2016_234_MOESM8_ESM.tif]
